# Pharmacological approaches in drug-resistant pediatric epilepsies caused by pathogenic variants in potassium channel genes

**DOI:** 10.3389/fncel.2024.1512365

**Published:** 2025-01-24

**Authors:** Ilaria Filareto, Ilaria Mosca, Elena Freri, Francesca Ragona, Laura Canafoglia, Roberta Solazzi, Barbara Castellotti, Giuliana Messina, Cinzia Gellera, Maria Virginia Soldovieri, Paolo Ambrosino, Maurizio Taglialatela, Jacopo C. DiFrancesco, Tiziana Granata

**Affiliations:** ^1^Department of Medical and Surgical Sciences of the Mothers, Children and Adults, University of Modena and Reggio Emilia, Modena, Italy; ^2^Department of Medicine and Health Sciences “Vincenzo Tiberio”, University of Molise, Campobasso, Italy; ^3^Department of Pediatric Neuroscience, member of the European Reference Network EPIcare, Fondazione IRCCS Istituto Neurologico Carlo Besta, Milan, Italy; ^4^Department of Epileptology, member of the European Reference Network EPIcare, Fondazione IRCCS Istituto Neurologico Carlo Besta, Milan, Italy; ^5^Unit of Medical Genetics and Neurogenetics, Fondazione IRCCS Istituto Neurologico Carlo Besta, Milan, Italy; ^6^Department of Science and Technology, University of Sannio, Benevento, Italy; ^7^Department of Neuroscience, University of Naples “Federico II”, Naples, Italy; ^8^Department of Neurology, Fondazione IRCCS S. Gerardo dei Tintori, Monza, Italy

**Keywords:** epilepsy, potassium channel, functional study, gabapentin (GBP), fluoxetine (FLX)

## Abstract

Variants in genes encoding for voltage-gated K^+^ (Kv) channels are frequent cause of drug-resistant pediatric epilepsies. Obtaining a molecular diagnosis gives the opportunity to assess the efficacy of pharmacological strategies based on *in vitro* features of mutant channels. In this retrospective observational study, we selected patients with drug-resistant pediatric epilepsies caused by variants in potassium channel encoding genes, followed at the Fondazione IRCCS Istituto Neurologico Carlo Besta of Milan, Italy. After the experimental characterization of variants’ functional properties in transiently transfected Chinese Hamster Ovary (CHO) cells, we identified drugs to be used as pharmacological approaches. We recruited six patients carrying different missense variants in four Kv channels (Kv7.2, Kv7.3, Kv3.1, and K_Na_1.1). *In vitro* experiments demonstrated that variants in Kv7 channels induced loss-of-function (LoF) effects, while those affecting Kv3.1 or K_Na_1.1 led to gain-of-function (GoF). Moreover, we found that the Kv7 channels activator gabapentin was able to revert the LoF effects caused by Kv7.2/Kv7.3 variants, and the potassium channel-blocker fluoxetine counteracted the GoF effects in Kv3.1 or K_Na_1.1 variants. According to experimental data, patients carrying Kv7 variants were treated with gabapentin. While this treatment resulted successful in two patients (#1, Kv7.2 G310S variant; #3, Kv7.3 V359L + Kv7.3 D542N), it resulted detrimental in the remaining case (#2, Kv7.2 D535E), requiring drug withdrawal. The application *in vivo* of fluoxetine to counteract GoF effects induced by Kv3.1 or K_Na_1.1 variants determined a significant reduction of both seizure frequency and behavior disturbances in patient #4 (Kv3.1 V425M), and in both subjects carrying K_Na_1.1 variants (#5, S937G and #6, R262Q). However, for the latter case, this drug was halted due to severe behavioral side effects. For most of the patients herein reported, pharmacological strategies, selected according to the *in vitro* functional properties of Kv-channels pathogenic variants, resulted in a significant improvement of both epileptic and cognitive features.

## Introduction

Pediatric-onset epilepsies are characterized by a variable course and prognosis, ranging from benign, self-limiting forms, to developmental and epileptic encephalopathies (DEEs), with drug-resistant seizures, and developmental delay (Zuberi et al., 2022; Scheffer et al., 2024).

Large-scale application of Next Generation Sequencing (NGS) techniques in the clinical practice often leads to the identification of pathogenic variants in many genes expressed in the brain, especially those coding for ion channels (Specchio and Curatolo, 2021; Castellotti et al., 2024). Among these, variants in genes encoding for voltage-gated K^+^ channels (Kv channels) subunits are frequently involved in the etiology of pediatric-onset epilepsies (Nappi et al., 2020). Variants in Kv channel genes cause phenotypes with variable severity, ranging from diseases with favorable evolution as self-limiting familial neonatal convulsions (SLFNS) due to KCNQ2 (Kv7.2) and KCNQ3 (Kv7.3) altered function (Singh et al., 2003; Maljevic and Lerche, 2014), to severe drug-resistant conditions termed KCNQ2- (Weckhuysen et al., 2012; Weckhuysen et al., 2013) or KCNT1- (encoding for K_Na_1.1 channels) related DEEs (Borlot et al., 2020; Morrison-Levy et al., 2021).

A genetic diagnosis may elucidate the pathogenic mechanism(s) of the disease, and provides the opportunity to identify pharmacological strategies able to counteract channel dysfunction observed *in vitro*, potentially improving the prognosis of patients (Truty et al., 2019; Møller et al., 2019; Bayat et al., 2021). Recent data indicate that 25 to 33% of cases of monogenic epilepsy may benefit from a personalized therapeutic approach. When initiated early in the disease’s clinical course, these strategies can lead to a reduction in seizures, mitigate side effects associated with extensive polytherapies, and yield significant economic benefits for the healthcare system, particularly in terms of treatment and hospitalization costs (Castellotti et al., 2024; Truty et al., 2019; Møller et al., 2019; Bayat et al., 2021).

In this study, we present a cohort of patients with drug-resistant epilepsy associated to pathogenic variants in potassium channel genes. These cases have been treated with pharmacological strategies selected on the basis of the functional characteristics (loss-of-function, LoF; gain-of-function, GoF) shown *in vitro* by the channels incorporating the disease-causing variants.

## Materials and methods

### Ethical approval and patients’ consent

This study received the ethical approval by the Institutional Review Board of the Fondazione IRCCS Istituto Neurologico Carlo Besta of Milan, Italy. Written informed consent for genetic analysis and use of anonymized clinical data for research purposes was obtained from all patients or guardians of participants. This research was performed in accordance with GCP and the ethical standards laid down in the 1964 Declaration of Helsinki.

### Patients’ recruitment

In this retrospective observational study, we selected patients with pediatric onset drug-resistant epilepsies caused by variants in potassium channel-encoding genes, consecutively recruited from a large cohort subjected to NGS genetic screening of target genes for epilepsy at the Fondazione IRCCS Istituto Neurologico Carlo Besta of Milan, Italy (Castellotti et al., 2024). Diagnosis of epilepsy was established according to the most recent criteria from the International League Against Epilepsy (ILAE) (Scheffer et al., 2017; Wirrell et al., 2022).

### Genetic screening

Following acquisition of study consent, genomic DNA was extracted from peripheral blood lymphocytes, according to standard procedures. Genetic analysis was assessed with a NGS approach as previously reported (Castellotti et al., 2024). Briefly, we designed target panels using Illumina design studio software^1^ and Agilent’s Sure Design tool.^2^ All the panels the following potassium channel coding genes: *KCNA1, KCNA2, KCNB1, KCNC1, KCND2, KCNE2, KCNH1, KCNH5, KCNH8, KCNJ10, KCNJ6, KCNK4, KCNMA1, KCNQ2, KCNQ3, KCNQ5, KCNT1, KCNT2, KCNV2, KCTD7, KDM6A, KIF1A, KIF2A, KIF5A, KIF5B,* and *KMT2D*.

According to ACMG criteria (Richards et al., 2015), the genetic variants of potassium channels passing the filtering process were classified as “pathogenic” (class V) or “likely pathogenic” (class IV); segregation analysis was performed by Sanger sequencing in both parents.

### Clinical data collection

We consulted medical records from a pseudo-anonymized database (available exclusively to the study researchers) and extracted the following information: genetic data [gene mutated, pathogenic variant(s), characteristics of variant(s), inheritance, ACMG class, functional effect]; clinical aspects [gender, age at diagnosis (months), neurological examination, neurodevelopmental delay/intellectual disability, behavioral features, other clinical aspects, brain MRI, age at seizure onset, epileptic syndrome, anti-seizure medications (ASMs), drug resistance, status epilepticus, EEG organization/background, epileptic activity].

We also collected the following information regarding pharmacological treatment with *in vitro* selected compounds (IVSCs): ASMs at IVSC start, IVSC compound, age of IVSC start, frequency of seizures at IVSC start, IVSC dose (mg/kg), IVSC in use, side effects of IVSC, frequency of seizure during IVSC treatment, improvement of other features, ASMs modification during IVSC treatment.

### Mutagenesis and heterologous expression of channel subunits

Variants herein reported were engineered by QuikChange site-directed mutagenesis (Agilent Technologies, Milan, Italy), as previously described (Miceli et al., 2015). In particular, the Kv7.2 D563E variant (numbering is based on the longest Kv7.2 transcript isoform a; accession number NM 172107.2; 872 amino acids) was introduced in a human KCNQ2 cDNA at the codon corresponding to the D535 residue in transcript isoform c (accession number: NM 004518.4; 844 amino acids), while the K_Na_1.1 R262Q variant was inserted in a plasmid containing the cDNA for a myc-DDK-tagged human isoform 2 (Q5JUK3–2) of KCNT1 (RC214820; Origene, Rockville, MD, USA).

Previously-studied variants in Kv7.2 [G310S (Soldovieri et al., 2020)], Kv7.3 [D542N and V359L (Ambrosino et al., 2018)], Kv3.1 [V425M (Ambrosino et al., 2023)], and K_Na_1.1 [S937G (Mosca et al., 2024)] were engineered with a similar strategy in plasmids containing the cDNA sequence for the corresponding wild-type Kv channels.

Chinese Hamster Ovary (CHO) cells were grown in 100-mm plastic Petri dishes in Dulbecco’s modified Eagle’s medium (DMEM) containing 10% fetal bovine serum (FBS), 2 mM L-glutamine, penicillin (50 U/mL) and streptomycin (50 μg/mL) in a humidified atmosphere at 37°C with 5% CO_2_.

Channel subunits were expressed in CHO cells by transient transfection using Lipofectamine (Thermofisher, Milan, Italy; 3.6 μg cDNA). An additional plasmid encoding for enhanced green fluorescent protein (EGFP; Clontech, Palo Alto, CA, USA; 0.4 μg cDNA) was used to identify transfected cells.

### Electrophysiological recordings

Patch-clamp recordings in the whole-cell configuration from transiently-transfected CHO cells, as well as data processing and analysis, were performed at RT 24 h after transfection as reported (Soldovieri et al., 2007; Rizzo et al., 2016). Briefly, for Kv7 currents cells were held at −80 mV and then depolarized for 1.5 s from −80 to +40 mV using an incremental pulse of 10 mV, followed by an isopotential pulse at 0 mV of 0.8 s; for K_Na_1.1 currents, cells were held at −80 mV and then depolarized for 0.6 s from −90 to +60 mV using an incremental pulse of 10 mV.

Current values recorded at the beginning of the 0 mV isopotential step (to record Kv7 currents) or at the end of the incremental pulse (to record K_Na_1.1 currents) were measured, normalized, and expressed as a function of each test voltage. The data were then fit to a Boltzmann distribution of the following form: *y = max/[1 + exp(V_½_ − V)/k]*, where *V* is the test potential, *V_½_* is the half-activation potential, and *k* is the slope factor.

To analyze activation kinetics, current traces recorded in response to incremental voltage steps were fitted to a single- or a double-exponential function of the following forms*: y = amp exp(−t/τ) or y = amp_FAST_ exp(−t/τ_FAST_) + amp_SLOW_ exp(−t/τ_SLOW_)*, respectively, where *amp_FAST_* and *amp_SLOW_* indicate the amplitude of the fast and slow exponential components, respectively, whereas *τ_FAST_* and *τ_SLOW_* indicate the time constants of these components. The relative contribution of the components in the total current was reported as *amp_RATIO_ = amp_FAST_/(amp_FAST_ + amp_SLOW_).*

Gabapentin (Sigma, Milan, Italy) was dissolved in distilled water, while fluoxetine (Sigma, Milan, Italy) in 0.01% DMSO. Both drugs were tested using ramp protocols from −80 to 0 mV in 3 s (for Kv7 currents) or from −90 to +60 mV in 1 s (for K_Na_1.1 currents), and perfused using a fast solution exchange system (<1 s), including a cFlow 8 flow controller attached to a cF-8VS 8-valve switching apparatus (Cell MicroControls, Norfolk, VA, USA), as previously described (Ambrosino et al., 2014). Drug-induced effects were calculated upon quantification of the area under the curve (AUC) of the current trace before (control) or after 1–3 minutes after drug application.

### Statistics

Each data point shown in Figures or in the text is the mean ± SEM of at least five distinct determinations, obtained in five distinct cells. Statistically significant differences were evaluated with the *Student’s t test* or with the ANOVA followed by the *Student–Newman–Keuls test*, when multiple groups were compared, with significance considered when *p* < 0.05.

## Results

### Study population

We identified six patients (five females) with drug-resistant epilepsy caused by pathogenic variants in genes coding for different potassium channels. Their genetic and clinical characteristics are summarized in Table 1.

**Table 1:** Genetic and clinical characteristics of the study population.

Patient #	Gender	Genetic characteristics					Clinical characteristics								
		Gene	Nucleotide variant	Protein change (running name)	Variant type	Inheritance pattern	Neurological features	Behavioral disorders	Intellectual disability	Age at seizure onset	Epileptic syndrome	EEG background	EEG epileptic activity	*Status epilepticus* (age)	ASMs used
1	F	*KCNQ2*	c.928G > A	p.Gly310Ser (G310S)	missense	*de novo*	spastic tetraparesis	no	severe	neonatal	early onset DEE	burst-suppression	multifocal	focal (neonatal)	PHT, PB, LEV, GVG, pyridoxine, CLB, ETS, VPA
2	F	*KCNQ2*	c.1689C > A	p.Asp563Glu(D535E)	missense	*de novo*	spastic tetraparesis	no	severe	neonatal	early onset DEE	diffuse slowing	multifocal	no	PHT, VPA, LTG, GVG, NZP, FBM, ACTH, TPM, CZP, CBD, GBP
3	F	*KCNQ3*	c.1624G > A (mother) c.1075G > T (father)	p.Asp542Asn (D542N)p.Val359Leu (V359L)	missense	compound heterozygosity	spastic tetraparesis	behavior disorder	severe	neonatal	early onset DEE	burst-suppression	multifocal	no	PB, GVG, pyridoxine
4	F	*KCNC1*	c.1273G > A	p.Val425Met (V425M)	missense	*de novo*	hypotonia, ataxia, apraxia	no	moderate	16 months	DEE with onset after 1 y.o.	poor organization	multifocal	no	VPA, CBZ, LTG, LEV
5	F	*KCNT1*	c.2809A > G	p.Ser937Gly (S937G)	missense	*de novo*	normal	aggressiveness, mood disorder	mild	36 months	focal epilepsy	diffuse slowing	multifocal	focal (11 years)	TPM, CBZ, OXC, NPZ, CLB, LTG
6	M	*KCNT1*	c.785G > A	p.Arg262Gln (R262Q)	missense	*de novo*	motor awkwardness	behavior disorder	severe	36 months	DEE with onset after 1 y.o.	poor organization	multifocal	no	LEV, CBZ, CLB, LTG, VPA, LCM, CZP, OXC, PB, BRV

ASMs: adrenocorticotropic hormone (ACTH), acetazolamide (ACZ), brivaracetam (BRV), cannabidiol (CBD), carbamazepine (CBZ), clobazam (CLB), clonazepam (CZP), diazepam (DZP), ethosuximide (ETS), felbamate (FBM), gabapentin (GBP), vigabatrin (GVG), lacosamide (LCM), levetiracetam (LEV), lamotrigine (LTG), methyl bromide (MB), nitrazepam (NZP), oxcarbazepine (OXC), phenobarbital (PB), phenytoin (PHT), topiramate (TPM), valproate (VPA).

In five patients (#1–4 and #6), the clinical picture was characterized by developmental and epileptic encephalopathy, with epilepsy beginning in the neonatal period or shortly thereafter. During the evolution of the disease, all patients developed moderate to severe intellectual disability with spastic tetraparesis, and experienced recurrent seizures refractory to several ASMs. The remaining case (#5) reported focal seizures with onset at the age of 3 years; the clinical course was characterized by recurrent focal seizures, refractory to ASMs, and evidence of mild intellectual disability, aggressiveness and mood disorder.

### Functional properties of mutant Kv channels

To investigate the functional properties of currents expressed by Kv channels incorporating the variants herein described, we performed electrophysiological *in vitro* experiments in mammalian cells transiently expressing distinct Kv channels.

Previous works showed that the Kv7.2 variant G310S (identified in patient #1), as well as those found in compound heterozygosity in Kv7.3 (V359L and D542N, identified in patient #3), prompt severe LoF effects, as these mutant subunits were non-functional (Soldovieri et al., 2020; Ambrosino et al., 2018; Mosca et al., 2022). Heteromeric Kv7.2/Kv7.3 channels mainly recapitulate the native M-current in adult neurons (Shah et al., 2002; Stewart et al., 2012); thus, the effects of these variants were also tested upon co-expression of mutant subunits with wild-type Kv7.2 and/or Kv7.3 subunits. The results obtained showed that, while Kv7.2 G310S subunits prompted dominant-negative effects on Kv7.2/Kv7.3 currents, haploinsufficiency was instead observed upon co-expression of either Kv7.3 variant with wild-type Kv7.2 subunits.

**Figure 1:**
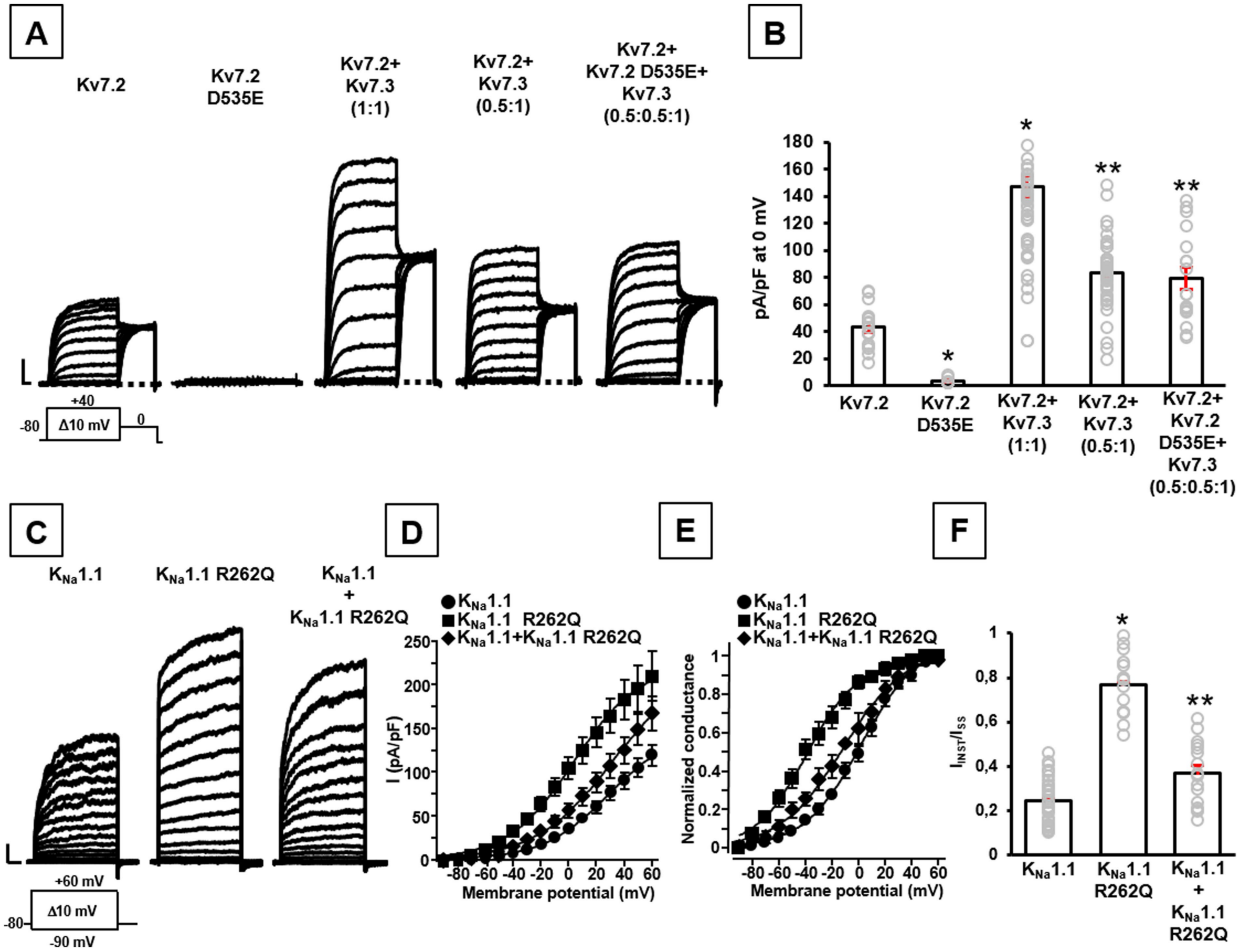
Functional properties of channels carrying the newly-identified Kv7.2 and KCNT1 variants. Representative family traces **(A)** and quantification of maximal current densities **(B)** recorded in CHO cells transiently expressing the indicated channels in response to the voltage protocol shown below the leftmost traces. Current scales: 500 pA; time scales: 200 ms. In panel **(B)**: *p < 0.05 versus Kv7.2, **p < 0.05 versus Kv7.2 + Kv7.3 (1:1). Representative current traces **(C)**, quantification of current densities **(D)**, conductance-voltage curves **(E)**, and quantification of I_INST_/I_SS_ ratios **(F)** recorded in CHO cells transiently expressing the indicated channels in response to the voltage protocol shown below the leftmost traces. Current scales: 500 pA; time scales: 100 ms. In panel **(F)**: *p < 0.05 versus K_Na_1.1. In panels **(B,F)**, gray symbols indicate single data points used for quantification.

Similarly, currents from Kv7.2 channels incorporating the novel D535E variant identified in patient #2 were undistinguishable from background (Figures 1A,B). When mutant subunits were co-expressed with wild-type channels (transfection ratio 1:1, 1.8 + 1.8 μg), a significant decrease of maximal currents was measured when compared to cells expressing only wild-type subunits (transfection ratio Kv7.2 channels+empty vector 1:1, 1.8 + 1.8 μg): in fact, current densities measured at 0 mV were 36.5 ± 5.5 or 16.9 ± 2.2 pA/pF in cells expressing Kv7.2 or Kv7.2 + Kv7.2 D535E subunits, respectively (*n* = 11–20; *p* < 0.05), thus suggesting dominant-negative effects prompted by mutant on wild-type Kv7.2 subunits. Furthermore, when Kv7.2 D535E subunits were co-expressed *in vitro* with Kv7.2 and Kv7.3 subunits at a transfection ratio (Kv7.2 + Kv7.2 D535E + Kv7.3,0.5:0.5:1) reproducing the genetic balance of the proband who was heterozygous for the KCNQ2 D535E variant, maximal currents were significantly reduced when compared to those measured in cells expressing Kv7.2 + Kv7.3 subunits, an experimental condition reproducing the genetic balance of a healthy control (Kv7.2 + Kv7.3, 1:1; Figures 1A,B). These results confirm the LoF effects prompted by the D535E variant. Moreover, currents measured in cells expressing Kv7.2 + Kv7.2 D535E + Kv7.3 subunits were identical to those observed in cells expressing Kv7.2 + Kv7.3 subunits (0.5:1 transfection ratio; Figures 1A,B), suggesting that in this heteromeric configuration mutant subunits prompt only haploinsufficiency (not dominant negative) effects. Finally, mutant subunits appear to alter activation kinetics of Kv7.2 + Kv7.3 currents: in fact, at −30 mV (where the maximal differences between Kv7.2 + Kv7.3 and Kv7.2 + Kv7.2 D535E + Kv7.3 channels were measured), τ_FAST_ were 116.3 ± 9.5 or 193.7 ± 21.6 ms (*n* = 18–50; *p* < 0.05), τ_SLOW_ were 535.2 ± 75.6 or 855.0 ± 108.3 ms (*n* = 11–28; *p* < 0.05), and amp_RATIO_ were 0.97 ± 0.02 or 0.81 ± 0.04 (*n* = 14–31; *p* < 0.05), for Kv7.2 + Kv7.3 or Kv7.2 + Kv7.2 D535E + Kv7.3 channels, respectively. These results suggest that heteromeric Kv7.2 + Kv7.3 channels activate at a significant slower rate in the presence of mutant subunits, thus confirming variant-induced LoF effects.

These results are similar to those previously reported for Kv7.3 V359L and Kv7.3 D542N variants carried in compound heterozygosity in patient #3 (Ambrosino et al., 2018): while homomeric Kv7.3 V359L or Kv7.3 D542N channels were non-functional *per se*, currents recoded in cells co-expressing Kv7.2 + Kv7.3 V359L + Kv7.3 D542N were significantly reduced when compared to wild-type Kv7.2 + Kv7.3 subunits, confirming variant-induced LoF effects. Also in this case, currents measured in cells expressing Kv7.2 + Kv7.3 V359L + Kv7.3 D542N subunits (transfection ratio 1:0.5:0.5) were identical to those measured upon co-expression of wild-type Kv7.2 + Kv7.3 subunits (1:0.5 transfection ratio), thus suggesting only haploinsufficiency effects in the presence of these mutant subunits.

By contrast, the herein reported K_Na_1.1 R262Q variant prompts a strong increase in maximal current density compared to wild-type channels (*n* = 15–34; *p* < 0.05; Figures 1C,D), as well as a leftward shift in their voltage-dependence of current activation (*V_½_* were 1.9 ± 2.3 mV or − 39.2 ± 2.5 mV for wild-type or mutant channels, respectively; *n* = 15–37; *p* < 0.05; *k* values were 21.3 ± 1.3 or 19.0 ± 1.8 mV/efold, respectively; *n* = 15–37; *p* > 0.05; Figure 1E). In addition, currents expressed by K_Na_1.1 R262Q channels showed a significant increase in the % of instantaneously-activated component (I_INST_) versus the total current (I_STEADY-STATE_ or I_SS_) compared to wild-type channels (*n* = 15–35; *p* < 0.05; Figure 1F); by contrast, the time-dependent component of maximal currents appeared slower in K_Na_1.1 R262Q mutant when compared to wild-type channels (*τ* values measured at +60 mV were 134.8 ± 6.6 ms or 226.9 ± 39.8 ms for wild-type or mutant channels, respectively; *n* = 12–28; *p* < 0.05). When mutant subunits were co-expressed with wild-type channels (transfection ratio 1:1), to reproduce the heterozygous state of the proband, voltage-dependence of current activation (*V_½_* was −11.5 ± 3.1 mV; *n* = 17; *p* < 0.05; Figure 1E) and the % of instantaneously-activated component (*n* = 16; *p* < 0.05 versus K_Na_1.1 and K_Na_1.1 R262Q homomeric channels; Figure 1F) resulted intermediate between homomeric wild-type and K_Na_1.1 R262Q mutant channels; by contrast, current density at +60 mV (*n* = 16; *p* > 0.05 versus K_Na_1.1 and K_Na_1.1 R262Q homomeric channels; Figures 1C,D) and the slope of the voltage-dependence activation curve (*k* value was 23.6 ± 1.9 mV/efold; *n* = 17; p > 0.05 versus K_Na_1.1 and K_Na_1.1 R262Q homomeric channels) were not significantly changed. Finally, current activation kinetics of the time-dependent component of maximal currents resulted indistinguishable from wild-type channels (τ value at +60 mV was 116.7 ± 7.8, *n* = 16; *p* > 0.05 versus K_Na_1.1; *p* < 0.05 versus K_Na_1.1 R262Q; Figure 1C): altogether, these results suggest that R262Q variant induces mainly GoF effects on wild-type channels, both in the homomeric and in heteromeric configurations.

Similarly, both previously-reported variants identified in Kv3.1 (V425M; Ambrosino et al., 2023) or K_Na_1.1 (S937G; Mosca et al., 2024) subunits prompted GoF effects when compared to the corresponding wild-type channels, in terms of increased current densities, leftwardly-shifted voltage-dependence of activation, absence of inactivation and/or slower deactivation kinetics. Qualitatively similar effects, although quantitatively smaller, were measured when wild-type and mutant subunits were simultaneously expressed to mimic the genetic balance of the proband (Ambrosino et al., 2023; Mosca et al., 2024).

### Pharmacological properties of mutant Kv channels

To counteract the distinct functional alterations prompted by the investigated Kv variants and considering that all reported patients had drug-resistant epilepsy, we searched for alternative treatments by studying *in vitro* the pharmacological sensitivity of Kv channels incorporating each variant to distinct drugs already approved for clinical use which were able to revert variant-induced changes in channel function.

**Figure 2:**
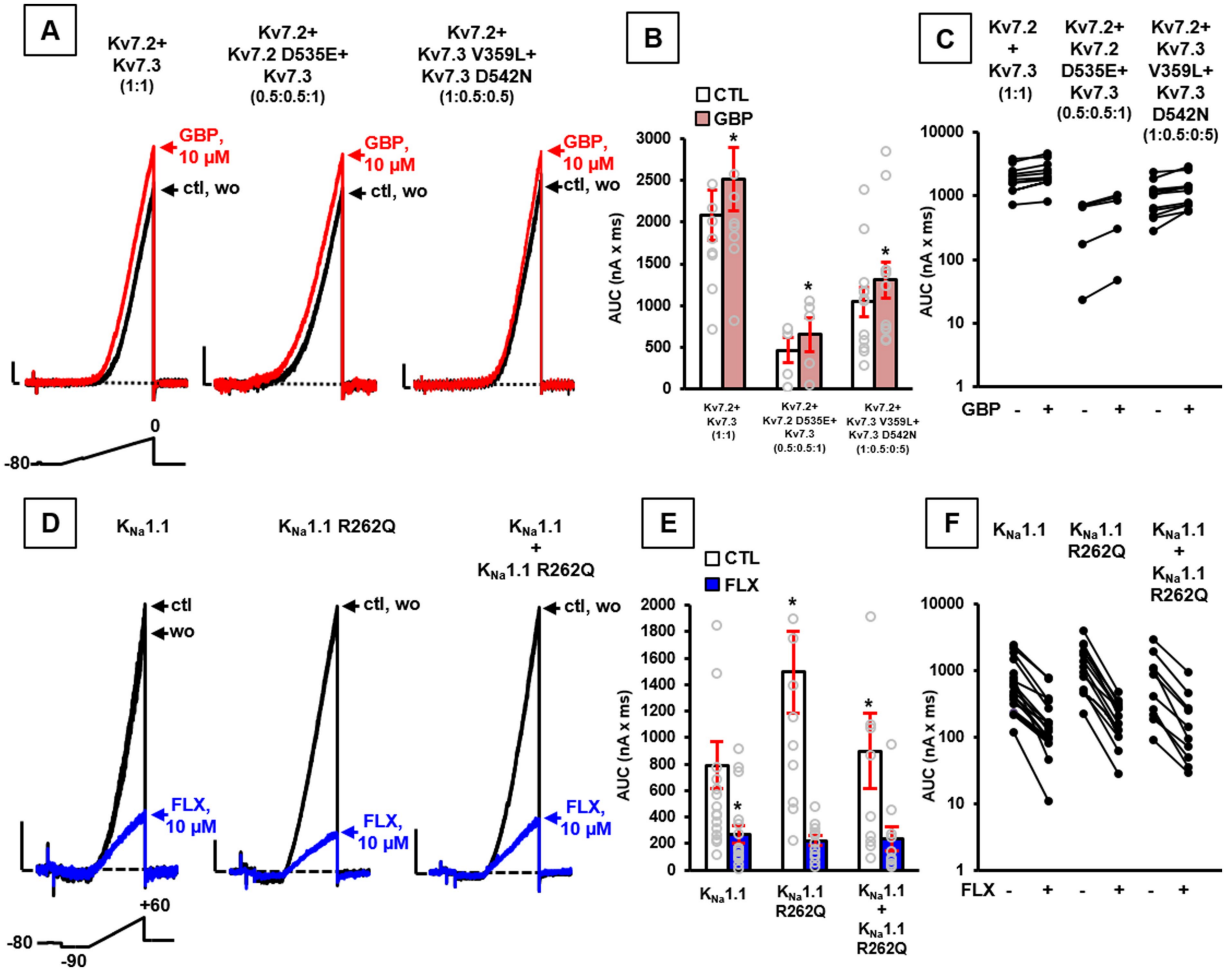
Pharmacological properties of channels incorporating Kv7.2, Kv7.3, or KCNT1 variants. Representative current traces **(A)**, quantification of AUC **(B)**, and **(C)** pre-post AUC values measured in single cells expressing the indicated channels at the transfection ratios specified in parenthesis upon exposure to the voltage protocol shown above the leftmost traces in control solution (ctl), upon exposure to 10 μM gabapentin (GBP), or upon drug washout (wo). Current scale: 200 pA; time scale: 200 ms. Representative currents traces **(D)**, quantification of AUC **(E)**, and **(F)** pre-post AUC values measured in single cells transiently expressing the indicated channels, upon application of the indicated voltage protocol, in control solution (ctl), upon exposure to 10 μM fluoxetine (FLX), or upon drug washout (wo), as indicated. Current scale: 500 pA; time scale: 200 ms. In panels **(B,E)**: *p < 0.05 versus each respective control.

According to the LoF effects described above in the presence of Kv7.2 or Kv7.3 variants, we performed additional patch-clamp recordings upon exposure to the anti-epileptic drug gabapentin (GBP), recently shown as a potent *in vitro* Kv7 activator (Manville and Abbott, 2018) and as an effective pharmacological treatment *in vivo* of patient #1 carrying the LoF-causing Kv7.2 G310S variant (Soldovieri et al., 2020). *In vitro* exposure to 10 μM GBP prompted a similar current increase in total currents (measured as the integral of the entire current trace, AUC) in cells expressing either wild-type Kv7.2 + Kv7.3, Kv7.2 + Kv7.2 D535E + Kv7.3, or Kv7.2 + Kv7.3 V359L + Kv7.3 D542N (Figures 2A–C), thus suggesting this drug as a rationale pharmacological alternative for probands carrying each of these variants.

On the contrary, since Kv3.1 V425M and K_Na_1.1 S937G variants both prompted GoF effects, a possible blocker of these currents was tested to counteract variant-induced functional alterations. As reported (Ambrosino et al., 2023; Mosca et al., 2024), the anti-depressant drug fluoxetine (FLX) showed the ability to block currents expressed by both Kv3.1 and K_Na_1.1 channels (also when carrying the Kv3.1 V425M and K_Na_1.1 S937G variants, respectively), with similar potency, thus suggesting the use of this specific drug in both probands carrying these variants.

Based on this evidence and according to GoF effects shown by K_Na_1.1 channels carrying the novel R262Q variant, 10 μM FLX was tested, revealing the ability of this drug to reduce current AUCs in CHO cells expressing wild-type K_Na_1.1 and K_Na_1.1 R262Q channels (Figures 2D–F; *n* = 12–17; *p* > 0.05).

### Treatment with *in vitro* selected compounds (IVSCs)

The presence of pathogenic variants in different potassium channel-encoding genes advocated the chance for targeted treatments. Different compounds were selected based on the patients’ specific gene variant causing distinct changes in channel function when studied in cellular models (Table 2). Based on the results of our functional studies, gabapentin was tested in three patients harboring LoF variants in KCNQ2 (patient #1 and #2) and KCNQ3 (patient #3), while fluoxetine was tested in those with GoF variants in KCNC1 (patient #4) and K_Na_1.1 (patients #5 and #6).

**Table 2:** *In vitro* selected compounds (IVSCs) for the treatment of patients with potassium channel-associated pediatric epilepsies.

Patient #	Pathogenic variant	*In vitro* functional properties	Drug tested *in vitro*	ASMs before IVSC start	Age of IVSC start (years, months)	Frequency of seizures before IVSC start	IVSC applied	IVSC last dose (mg/kg)	IVSC last plasma level	IVSC side effects	Follow-up (years, months)/Notes	Ongoing ASMs	Ongoing frequency of seizure	EEG modification	Modification of other features
1	Kv7.2-G310S	LoF	GBP	VPA, LEV	7,7	monthly	GBP	26	5.7 mg/L	None	6,2	GPB, VPA. LEV suspended	seizure free	better background activity, decrease of epileptiform activity	improved interaction and communication
2	Kv7.2-D535E	LoF	GBP	TPM, CZP	6,2	monthly seizures; daily spasms	GBP	9.6	N.A.	Increase of seizure frequency	3,10 / GBP suspended after 1 month, introduced CBZ	CBZ	seizure free	unchanged	improvement of attention and participation
3	Kv7.3-V359L + K v7.3-D542N	LoF	GBP	GVG	10,3	daily	GBP	28	3.4 mg/L	None	3,4	GBP. GVG suspended	seizure free	reduction of epileptiform activity	reduction of irritability, improved head-trunk control, walking with support, interaction with environment
4	Kv3.1-V425M	GoF	FLX	VPA, LEV	4,5	febrile seizures in cluster	FLX	0.24	137 ng/mL	None	5,6	FLX, LEV. VPA suspended	seizure free	better background activity, decrease of epileptiform activity	improvement in behavior, participation in school and social activities
5	K_Na_1.1 S937G	GoF	FLX	OXC, CZP	18,3	daily	FLX	1.2	412.6 ng/mL	None	3,1	FLX, OXC, CZP	seizure free	normal background, disappearance of epileptic activity	active social life
6	K_Na_1.1 R262Q	GoF	FLX	OXC, VPA, CBD	5,0	daily	FLX	0.8	447.5 ng/mL	Worsening of behavior disorder	4,5 / FLX suspended after 10 months	VNS, VPA, OXC, CZP	persistent seizures	unchanged	unchanged

ASMs: cannabidiol (CBD), carbamazepine (CBZ), clonazepam (CZP), fluoxetine (FLX), gabapentin (GBP), vigabatrin (GVG), levetiracetam (LEV), oxcarbazepine (OXC), topiramate (TPM), vagus nerve stimulation (VNS), valproate (VPA).

We previously demonstrated that the exposure to the Kv7 activator gabapentin can potentiate channel function in the presence of the Kv7.2 G310S variant carried by patient #1 (Soldovieri et al., 2020). Gabapentin was introduced and titrated up to 28 mg/kg/daily, reaching a plasma level of 3.4 mg/L. This treatment resulted significantly and persistently effective. During the subsequent six years of follow-up, gabapentin was well tolerated (daily dose is currently 26 mg/kg, with a drug plasma level of 5.7 mg/L), seizures did not recur, and the girl significantly improved in interaction and communication. Moreover, the EEG showed a marked decrease of epileptiform discharges.

Based on the successful treatment of patient #1, together with *in vitro* evidence showing the ability of gabapentin in recovering the functional deficit due to the Kv7.2 D535E variant (Figures 2A–C), we also tested the effect of this compound in patient #2. Unfortunately, treatment with gabapentin up to 9.6 mg/kg/daily induced worsening of seizure frequency, and the drug had to be withdrawn. Carbamazepine, known to be effective in patients carrying Kv7.2 variants (Falsaperla et al., 2023; Dilena et al., 2022; Pisano et al., 2015; Sands et al., 2016), was than titrated up to 18 mg/kg/daily (plasma level 7 μg/mL), with full control of seizures. During the following three years of follow up, topiramate and clonazepam were withdrawn, no seizure recurred and we observed a mild improvement in attention and participation.

According to the *in vitro* results reported in (Figures 2A–C), gabapentin was also applied in patient #3, carrying the two LoF variants Kv7.3 V359L and Kv7.3 D542N. This drug, gradually increased up to 28 mg/kg/daily reaching plasma level 3.4 mg/L, led to the rapid disappearance of tonic spasms, and allowed withdrawing vigabatrin four months later. The EEG showed a marked decrease of epileptic activity, and no seizure recurred in the following three years of follow-up. Furthermore, postural control of head and trunk slightly improved, so that the child is now able to drive her own wheelchair.

On the other hand, for the treatment of patients #4, #5 and #6, carrying GoF variants in Kv3.1 or K_Na_1.1 potassium channels, we tested *in vivo* the effect of the potassium channel-blocker fluoxetine.

In particular, patient #4 did not respond to numerous ASMs and developed drug-resistant epilepsy. *In vitro* incubation with fluoxetine of cells expressing the Kv3.1 V425M variant resulted in the reduction of the current produced by the mutated channels (Ambrosino et al., 2023). Treatment with this compound at 0.26 mg/kg (plasma level 171.3 ng/mL) resulted in the complete control of seizures, and allowed to suspend valproate. During the five and a half years of follow-up (current dose 0.24 mg/kg, plasma level 137 ng/mL), the patient did not experience seizures, and a noticeable improvement in behavioral aspects with greater participation in school activities and social interaction was observed. Moreover, serial EEG traces showed significant reduction of epileptic activity.

Fluoxetine was also used for the treatment of patient #5, carrying the K_Na_1.1 S937G variant. This drug at the dosage of 1.2 mg/kg (plasma level 412.6 ng/mL) led to the complete control of epileptic seizures, along with significant improvement of the EEG pattern (Mosca et al., 2024). The patient has been under treatment with fluoxetine for over three years, no seizures have occurred during follow-up, and the patient has an active social life. However, despite the complete control of seizures, the girl refused to reduce the ASMs oxcarbazepine and clonazepam.

According to *in vitro* data demonstrating the ability of fluoxetine in recovering the functional defect prompted by the K_Na_1.1 R262Q variant (Figures 2D–F), we treated with this drug also patient #6. Fluoxetine was gradually increased up to 0.8 mg/kg, reaching a plasma level of 447.5 ng/mL, leading to disappearance of seizures in wakefulness and their reduction during sleep. However, along with improvement of the epileptic symptoms, we observed a progressive worsening of behavior disorder, including marked agitation, akathisia, and the emergence of compulsions and mannerisms. Suspecting a possible side effect of fluoxetine, this was discontinued after ten months, leading to the regression of behavioral disorders, but with reappearance of high-frequency seizures during wakefulness.

## Discussion

In this study, we report the results of the treatment of patients with drug-resistant pediatric epilepsies caused by different missense variants in four voltage-dependent potassium channels (Kv7.2, Kv7.3, Kv3.1, and K_Na_1.1) with drugs selected for their ability to counteract mutation-induced channel dysfunction observed *in vitro*.

In order to understand pathogenic mechanisms triggered by each variant, we first studied their functional effects in transiently transfected mammalian cells by means of patch-clamp electrophysiological recordings. Our data showed that variants identified in Kv7.2 or Kv7.3 induced LoF effects, while those affecting Kv3.1 or K_Na_1.1 led to GoF. Then, we tested *in vitro* compounds able to potentiate Kv7.2/Kv7.3 or block Kv3.1/K_Na_1.1 currents, identifying gabapentin and fluoxetine, respectively, as putative tailored drugs.

Among substances known to modulate Kv channels, we focused on those able to pass through the blood–brain barrier and with a well-known direct action on the central nervous system. Notably, while gabapentin is a widely used ASM, generally well tolerated by most patients, population studies demonstrate that selective serotonin reuptake inhibitors (SSRIs) increase the risk of developing epilepsy (Chou et al., 2017; Christensen et al., 2019). However, both these compounds have different molecular targets. While gabapentin is a known modulator of Ca^2+^ channels (Gee et al., 1996), antidepressant effects of fluoxetine are mainly attributed to the selective inhibition of serotonin reuptake (Ampuero et al., 2024). Moreover, evidence shows that serotonin also plays a role in neural excitability, epileptogenesis, and seizure propagation. Experimental data from animal models demonstrate that agents able to increase extracellular levels of serotonin, as SSRIs, can inhibit focal and generalized seizures (Alper et al., 2007; Richerson and Buchanan, 2011; Bagdy et al., 2007).

According to these data, since LoF effects were detected for all Kv7 variants herein reported, we searched for activators of these channels. As an alternative to retigabine, a well-known selective Kv7 activators unavailable on the market since 2017,^3^ we focused on gabapentin, recently emerged *in vitro* as a potent enhancer of Kv7 currents (Manville and Abbott, 2018). This compound already resulted effective in proband #1 harboring the G310S variant in Kv7.2 (Soldovieri et al., 2020). Moreover, gabapentin was also able to revert *in vitro* the LoF effects of the Kv7 variants found in probands #2 and #3.

However, contrasting results were observed. In fact, while gabapentin resulted successful in proband #3 (carrying Kv7.3 V359L + Kv7.3 D542N variants), it was detrimental in patient #2 (carrying the Kv7.2 D535E variant), who experienced increased seizure frequency, requiring gabapentin withdrawal after one month. In this case, seizure freedom was achieved with the introduction of carbamazepine, known to be effective in Kv7-related epilepsies (Pisano et al., 2015; Sands et al., 2016).

Variability in clinical response to the same compound might be explained by several factors. Among these, the age of patients at treatment initiation, the channel region affected by the variant, together with its functional and pharmacological effects, and their interaction of the newly-introduced drug with concomitant ASMs, are the most relevant. Though, in the cohort herein reported, we were not able to identify such factors. In particular, probands carrying Kv7 variants featured similar clinical phenotypes and ages at seizure onset. Furthermore, cases #2 and #3 exhibited variants at paralogous positions in Kv7.2 (D535) and Kv7.3 (D542), respectively, suggesting that their localization is unlike to play a major role in the heterogeneity of clinical responses. We could speculate that the different ASMs taken by patients may have led to distinct cellular adaptation phenomena, resulting in divergent therapeutic effects. However, the knowledge available to date does not allow to draw any certain conclusions.

On the contrary, to counteract GoF effects induced by Kv3.1 or K_Na_1.1 variants, we selected drugs able to block these currents. While the large majority of Kv3.1 variants associated with epilepsy present LoF pathogenic mechanisms (Cameron et al., 2019), those with GoF were reported only recently (Clatot et al., 2022), and data on their treatment are lacking.

GoF variants in K_Na_1.1 channels are frequent causes of DEE with drug-resistant epilepsy (Bonardi et al., 2021). Although the antiarrhythmic quinidine, a low potency blocker of K_Na_1.1 channels (Yang et al., 2006), can be considered a therapeutic opportunity for these cases (Milligan et al., 2014), its clinical use is hampered by important cardiotoxic effects (Liu et al., 2023). Indeed, variable clinical responses were reported in several case series: while some patients present a good, albeit often transient, response to quinidine, others do not respond, and different strategies become necessary (Sills, 2023; Abdelnour et al., 2018; Dilena et al., 2018; McTague et al., 2018; Xu et al., 2022; Mullen et al., 2018). In addition, the amino acid residue 937 is located in a region of the channel close to others (R929, A934, and K947) whose pathogenic variants appear to be resistant to quinidine treatment (Fitzgerald et al., 2019).

It is known that the widely used anti-depressant drug fluoxetine (Sung et al., 2008), and even more by its major *in vivo* metabolite norfluoxetine (Choi et al., 2001), is able to block currents sustained by Kv3.1 channels. Therefore, we tested this compound *in vitro* and demonstrated its effect in contrasting the enhanced channel activity caused by the V425M variant carried by patient #4, reporting reduction of both seizure frequency and behavior disturbances (Ambrosino et al., 2023). Fluoxetine was also successfully given to patient #5, following *in vitro* demonstration of its ability to inhibit currents expressed by both wild-type channels and those carrying the GoF K_Na_1.1 S937G variant found in this patient (Mosca et al., 2024). The positive effect on seizure frequency was also observed in patient #6 (carrying the R262Q variant in K_Na_1.1); however, the drug was halted because of severe behavioral side effects. In this case, the anti-seizure effect of fluoxetine was further confirmed by the reappearance of seizures at high frequency following drug withdrawal.

Overall, our data indicate that for most of cases herein reported, the implementation of pharmacological strategies selected on the basis of drug’s ability to counteract mutation-induced channel dysfunction *in vitro* resulted in a significant clinical improvement, including seizure control and amelioration of EEG patterns. In addition to the epileptic phenotype, we observed improvement in the cognitive and behavioral domains. Furthermore, in some cases, previously used conventional ASMs were discontinued, without clinical worsening, in particular without increase in seizure frequency. These findings are in agreement with the concept of ideal therapies, which should be both effective and well tolerated by patients. As further evidence of effectiveness and tolerability of this approach, is the fact that none of the responsive patient has yet discontinued the newly-introduced drug.

### Limitations

The main limitation of this work derives from the small number of patients recruited. However, these are extremely rare conditions and patients come from a single center. Notably, we included in this study only cases with drug-resistant epilepsy caused by KCN variants who required therapeutic approaches selected on the basis of drug’s ability to counteract mutation-induced channel dysfunction *in vitro*, while we excluded those well controlled by conventional ASMs.

## Conclusions

The data presented here further confirm the importance of an early and comprehensive clinical-genetic characterization of patients with drug-resistant pediatric-onset epilepsy. The widespread availability of advanced NGS techniques allows the identification of an increasing number of patients with causative variants. Electrophysiological recordings in transiently transfected mammalian cells are a useful approach to clarify the functional properties of causative variants. Especially for potassium channelopathies, these studies can identify the pathogenic mechanisms of the disease, mainly LoF or GoF effects. In addition being crucial for several clinical aspects, including prognostication and phenotype stratification, experimental data can guide the implementation of treatments using drugs selected on the basis of their ability to counteract the functional defects specific for the different pathogenic Kv channel variants.

## Data Availability

The raw data supporting the conclusions of this article will be made available by the authors, without undue reservation.

## Footnotes

^1^ https://designstudio.illumina.com

^2^ https://earray.chem.agilent.com/suredesign/

^3^ https://www.ilae.org/files/dmfile/GSK_RetigabineTrobalt-Reminder.pdf
